# Effect of changes in green spaces on mental health in older adults: a fixed effects analysis

**DOI:** 10.1136/jech-2019-212704

**Published:** 2019-10-19

**Authors:** J Mark Noordzij, Marielle A Beenackers, Joost Oude Groeniger, Frank J Van Lenthe

**Affiliations:** Public Health, Erasmus Medical Center, Rotterdam, The Netherlands

**Keywords:** mental health, urban health, geographical information systems, urban development, ageing

## Abstract

**Background:**

Urban green spaces have been linked to different health benefits, but longitudinal studies on the effect of green spaces on mental health are sparse and evidence often inconclusive. Our objective was to study the effect of changes in green spaces in the residential environment on changes in mental health using data with 10 years of follow-up (2004–2014).

**Methods:**

Data from 3175 Dutch adults were linked to accessibility and availability measures of green spaces at three time points (2004/2011/2014). Mental health was measured with the Mental Health Inventory-5. Fixed effects analyses were performed to assess the effect of changes in green spaces on mental health.

**Results:**

Cross-sectional analysis of baseline data showed significant associations between Euclidean distances to the nearest green space and mental health, with an increase of 100 m correlating with a lower mental health score of approximately 0.5 (95% CI −0.87 to −0.12) on a 0–100 scale. Fixed effects models showed no evidence for associations between changes in green spaces and changes in mental health both for the entire sample as well as for those that did not relocate during follow-up.

**Conclusions:**

Despite observed cross-sectional correlations between the accessibility of green space in the residential environment and mental health, no evidence was found for an association between changes in green spaces and changes in mental health. If mental health and green spaces are indeed causally linked, then changes in green spaces in the Eindhoven area between 2004 and 2014 are not enough to produce a significant effect.

## Introduction

From 1990 to 2010, the burden of mental health increased by 38%, an increase mostly attributable to population growth and ageing. Major depressive disorder, a common mental disorder in older age, is the leading cause of disability-adjusted life years (DALYs) and the fourth leading contributor to the global burden of disease worldwide.^1^ Mental disorders in old age lead to impairments in the ability to function socially, decreased quality of life, and increased risk of health problems and comorbidities. They carry substantial social and economic impacts on families and societies, imposing a burden on health and social care services.^1^ Decades of research have documented the higher risk of mental disorders among those living in urban versus rural areas.^2^ Global urbanisation trends have led to more and more people living in cities, with urbanisation affecting the whole world.^3^ This situation of planetary urbanisation means that the urban environment has become a key site for the implementation of prevention and early identification policies on the trajectories of ageing and mental well-being.

Within the context of an increasingly urbanising world, contact with natural environments may play an important role in improving mental health. A review by the WHO indicated mental health as being one of the most important factors influenced by urban green spaces.^4^ Other studies have shown that individuals living in urban areas with more green space have a reduced level of stress and improved well-being compared with controls with poorer availability of green space.^5 6^ However, the mechanisms linking green spaces to mental health appear to be complex, leading to much discussion on underlying pathways. Psychoevolutionary theories suggest that mental health can be influenced through restorative functions of natural environments. Views of, or interaction with, nature can reduce stress,^7 8^ or involuntary attention given to stimuli from nature can aid in performing cognitively demanding tasks.^9 10^ Other mechanisms include green spaces supporting physical activity, stimulating social interactions and reducing exposure to harmful environmental stressors.^11 12^

While a substantial number of studies present significant associations between green spaces and mental health, they are often based on cross-sectional data.^5 13^ Thus, causality cannot be established, putting into question whether increasing the amount of green spaces leads to better mental health. The evidence of long-term mental health benefits of urban green spaces seems to be inconsistent at best, as many studies are hampered by weak statistical associations, or failure to exclude confounding, bias or reverse causality.^14 15^ Longitudinal studies that do assess how green space and mental health relate over time provide evidence that the impact of green spaces on mental health can vary across the life course,^16^ or find little to no impact at all.^17^ This further raises questions about the strength and robustness of cross-sectional findings relating mental health to green spaces.

An attractive method to address these concerns comes with the use of fixed effects models that rely on within-individual changes. This method eliminates the effects of time-invariant confounding variables as long as they remain stable over time (ie, they are ‘fixed’).^18^ A UK study that used this approach found that ‘respondents in areas with more green space experienced significantly lower mental distress and significantly higher well-being’.^6^ However, a complicating factor of these models is that the method requires multiple measurements. While individual-level longitudinal outcome data are becoming increasingly available, individual-level longitudinal exposure data are still rare. Longitudinal studies therefore commonly rely on neighbourhood-level data or small-area statistics and link those exposures to individual-level outcomes.^6^ The problems associated with such linkages have been described in detail.^19 20^ The present study, however, is able to circumvent these problems by using a harmonised, longitudinal geographical information system (GIS) database to generate individual-level green space exposure data. Using individual-level exposures helps to circumvent methodological problems of area-level data and will strengthen the evidence base of the effects of green spaces on mental health. To the best of our knowledge, no studies to date exist that use fixed effects models to investigate how green space and mental health of older adults relate over time using both individual-level exposures and outcomes.

Our present study links individual urban green space exposures to mental health outcomes from cohort data with 10 years of follow-up. We first describe group-level associations deduced from a cross-sectional analysis of the baseline data. Second, we explore within-subject changes with a fixed effects model. Lastly, we estimate within-subject changes among participants who did not relocate during follow-up. Sensitivity analyses were performed using random effects models that explore variation between individuals, and on data on the amount of green space within the residential environment.

## Methods

### Study population

**Figure 1:**
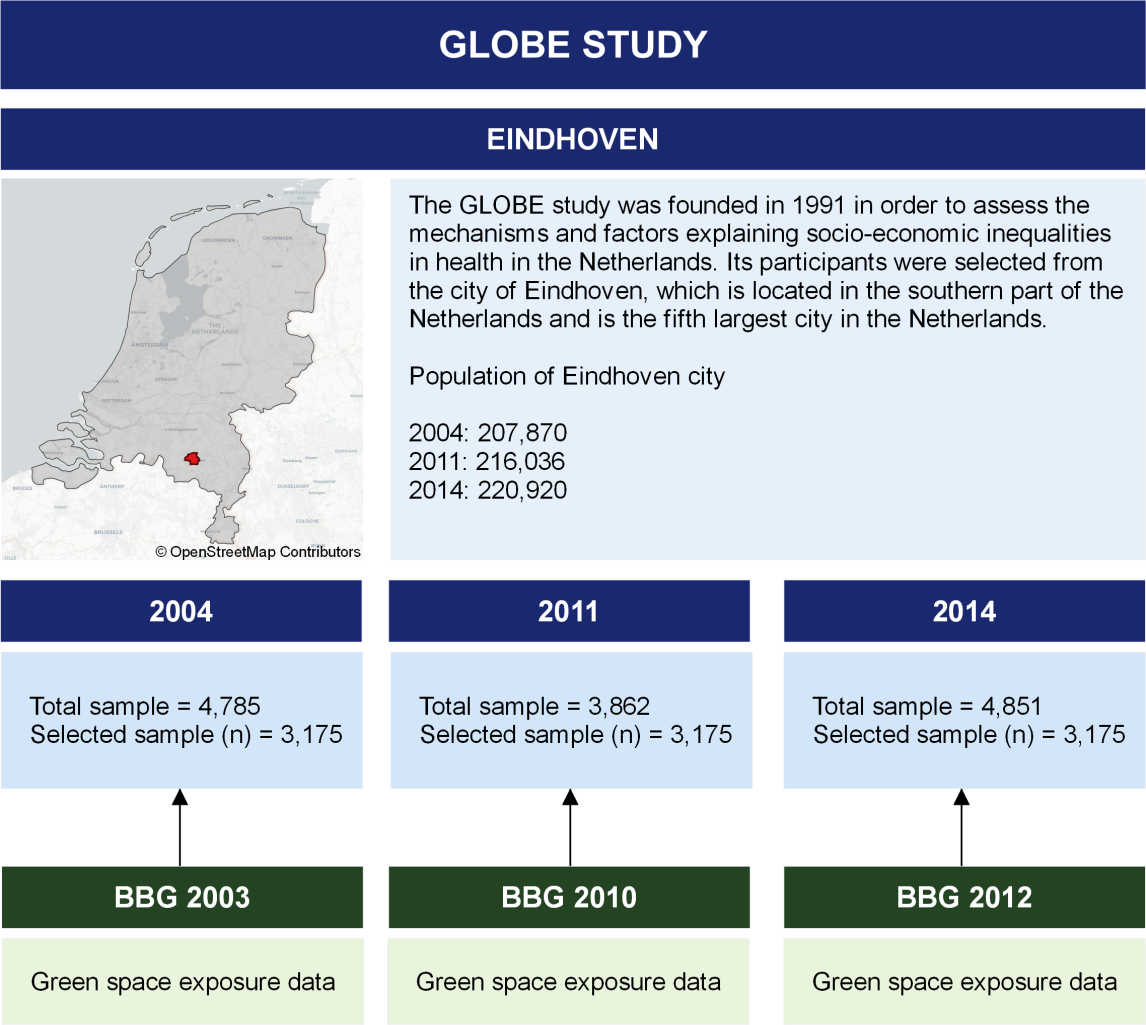
Study overview of the GLOBE study (Gezondheid en Levens Omstandigheden van de Bevolking van Eindhoven en omstreken) and the Eindhoven area. BBG, Bestand Bodemgebruik.

Data were obtained from GLOBE (Gezondheid en Levens Omstandigheden van de Bevolking van Eindhoven en omstreken), a prospective cohort study on the role of living conditions for health in the Netherlands. The 2004 sample of GLOBE participants was selected for the analyses (n=4785) with follow-up data collected for the years 2011 and 2014 (figure 1). The sample consisted mainly of older adults living in the city of Eindhoven and surrounding areas. Additional details of the GLOBE study can be found elsewhere.^21^ The residential addresses of these respondents were geocoded using the geographical software package QGIS^22^ and a geocoding plug-in developed by the Dutch National Spatial Data Infrastructure ‘Publieke Dienstverlening Op de Kaart’ (PDOK).^23^ To maintain respondent privacy, addresses were extracted and geocoded using a process previously described.^24 25^ Additional questionnaires were administered in 2011 and 2014. Respondents who only participated in 1 year were excluded (33%), resulting in a final sample of 3175 respondents. Movement to different addresses between follow-up years was recorded.

### Exposure measures of green space

Exposure measures of this study were obtained using the data set ‘Bestand Bodemgebruik’ (BBG), which is maintained by Statistics Netherlands.^26^ The BBG database is a harmonised data set based on ‘Top10NL’ digital 1:10 000 topographic maps provided by the Dutch mapping agency Kadaster.^27^ The harmonisation of the BBG data ensures that observed changes are representative of actual changes in the environment and not related to changes in GIS processing or methodology. Extensive land classification data were used to locate categories of green spaces based on previous research in the Netherlands using similar data^28^ (online supplementary appendix 1). The classifications were subsequently divided into four categories: (1) green spaces, (2) green and blue spaces, (3) green and agricultural spaces, and (4) green, blue and agricultural spaces. Accessibility measures were calculated as the Euclidean distance from the participant’s residential address to the nearest point on the boundary of a green space for each participant at each time point using QGIS. Availability of green space was calculated based on the amount of green spaces within the Euclidean buffers of 300 m, 500 m and 1000 m around the residential address. Sensitivity analyses were performed on respondents from Eindhoven inner city and respondents from the Eindhoven city region (figure 1). GLOBE cohort data from each wave were linked to geographical data from the preceding year, keeping in line with an appropriate chronology of exposure preceding outcome (figure 1). Unfortunately, BBG data were not available for 2013, so 2014 outcome data were linked to exposure data from 2012.

10.1136/jech-2019-212704.supp1 Supplementary data

### Outcome measures of mental health

Mental health was assessed using the five-item version of the ‘mental health inventory’ (MHI-5). MHI-5 is a validated questionnaire that asks respondents how their mental health was over the last 4 weeks.^29 30^ It consists of the following five questions: (1) *‘Have you felt so down in the dumps that nothing could cheer you up?’*, (2) *‘Have you felt downhearted and blue?’*, (3) *‘Have you been a happy person?’*, (4) *‘Have you been a very nervous person?’*, and (5) *‘Have you felt calm and peaceful?’*. Each question has six possible responses ranging from ‘all the time’ to ‘none of the time’; the third and fifth questions were reverse-coded. A total mental health score was calculated by taking the mean of the five items and transforming it to 100-point scale to improve interpretation (a higher score indicates better mental health).^29 30^ Cronbach’s alpha for the MHI-5 scale was 0.85. Participants had to answer at least three out of five questions to be included.

### Covariates

Marital status (married/partnership, not married, divorced, widowed), annual household income (monthly; <€1200, €1200–€1800, €1800–€2600, >€2600) and employment status (employed, unemployed, retired, non-employed) were included as relevant time-varying confounders. All covariates were measured at all three time points, capturing changes that occurred in the 10-year period. Time-invariant characteristics (as measured in 2004) that were included in the cross-sectional analyses include age, gender (male, female), place of birth (the Netherlands, elsewhere) and education classified using the International Standard Classification of Education (ISCED) (lowest=ISCED 0–1, low=ISCED 2, middle=ISCED 3–4, high=ISCED 5–7).^31^

### Statistical analyses

Missing data on covariates were handled via multiple imputation using data on the variables listed above, as well as self-rated health (excellent, very good, good, fair, poor), smoking (yes, no), home ownership (rental, owner), financial stress (no, some, yes) and body mass index.^32^ Missing data ranged from 0% on the exposures to 36% on income (online supplementary appendix 3). First, cross-sectional analyses were performed on baseline data from 2004. Associations between exposure and outcome were explored with linear regression models adjusted for age, age squared, gender, place of birth, education, marital status, income and employment. Second, fixed effects models were used to estimate the relationship between within-person changes in the distance to the nearest green space and within-person changes in mental health. Two fixed effects models were applied: a linear regression model controlling for time only, and an adjusted model with additional controls for time-varying characteristics of marital status, employment and income. The following model was used for the analyses:

10.1136/jech-2019-212704.supp3 Supplementary data

$${MentalHealth}_{it}=\mu_{t}+\beta_{1}{GreenSpace}_{it}+\beta_{2}x_{it}+\alpha_{i}+\epsilon_{it}$$

whereby ${MentalHealth}_{it}$ indicates the total mental health score for individual *i* at time *t*, $\mu_{t}$ accounts for time effects that are fixed for all individuals, ${GreenSpace}_{it}$ represents the green space exposure measure (ie, the distance to the nearest green space or the area within the designated buffer), $x_{it}$ is a vector of time-varying control regressors, $\alpha_{i}$ controls for time-invariant personal characteristics, while $\epsilon_{it}$ is the error term. The fixed effects analyses were performed first on all available data, and second on data restricted to participants who did not relocate during follow-up. Robust SEs were used to account for non-independence clustering at the individual level. Sensitivity analyses were performed using random effects models that explore variation between individuals. All analyses were performed using Stata V.15.^33^

## Results

At baseline (2004; table 1) the mean age was 53 years, and 55.5% of the participants were women. On average, the total mental health score of respondents was 73.2 on a 0–100 scale. The distance to the nearest green space ranged from 163 m to 193 m on average between different green space categories. The amount of green space ranged from an average amount of 3.46 hectares in the smallest buffer (300 m) to 47.75 hectares in the largest (1000 m).

**Table 1:** Description of the study population at baseline (2004, n=3175)

Variables	Mean (SD)/%
Exposures	
Distance to nearest green space, m	193 (139)
Distance to nearest green or blue space, m	186 (136)
Distance to nearest green or agricultural space, m	169 (129)
Distance to nearest green, blue or agricultural space, m	164 (126)
Amount of green spaces within 300 m buffers, hectares	3.46 (3.01)
Amount of green spaces within 500 m buffers, hectares	9.66 (7.70)
Amount of green spaces within 1000 m buffers, hectares	47.75 (27.61)
Outcome	
Total mental health score (MHI-5)	73.2 (15.7)
Time-fixed characteristics	
Male, %	44.5
Born in the Netherlands, %	93.0
Educational level, %	
High	31.3
Middle	24.7
Low	35.1
Lowest	8.9
Time-varying characteristics	
Age, mean (SD)	53 (13)
Marital status, %	
Married/partnership	75.6
Unmarried	12.1
Divorced	6.9
Widowed	5.4
Employment, %	
Employed	50.3
Unemployed	7.4
Retired	25.7
Non-employed	16.6
Household income, %	
<€1200	10.4
€1200–€1800	20.1
€1800–€2600	27.9
€2600–€4000	29.1
>€4000	12.5

MHI-5, Mental Health Inventory-5.

Linear regression models applied to cross-sectional data from 2004 showed significant associations between the distance to the nearest green space and the total mental health score for all green space categories (table 2). On average, the total mental health score declined with 0.49 (95% CI −0.87 to −0.12) to 0.55 (95% CI −0.96 to −0.13) points when the distance to the nearest green space was extended by 100 m. Sensitivity analyses showed that these results were only observed among respondents within the suburban areas and not among respondents within the inner city (online supplementary appendix 2). Applied random effects models showed similar effect directions, but effect sizes were attenuated greatly (online supplementary appendix 2). The amount of green space in hectares within buffers was not significantly associated with the total mental health score (table 2).

10.1136/jech-2019-212704.supp2 Supplementary data

**Table 2:** Linear regression models regressing total mental health on the distance to the nearest green, blue and agricultural spaces using cross-sectional data from 2004 (n=3175)*

	β	95% CI	P value
Distance to nearest green space (100 m) Total mental health score	−0.494	−0.865 to −0.122	0.009
Distance to nearest green or blue space (100 m) Total mental health score	−0.584	−0.965 to −0.204	0.003
Distance to nearest green or agricultural green space (100 m) Total mental health score	−0.445	−0.846 to −0.043	0.030
Distance to nearest green, blue or agricultural green space (100 m) Total mental health score	−0.547	−0.960 to −0.134	0.010
Amount of green spaces within 300 m buffers (hectares) Total mental health score	0.120	−0.071 to 0.311	0.219
Amount of green spaces within 500 m buffers (hectares) Total mental health score	0.055	−0.012 to 0.123	0.109
Amount of green spaces within 1000 m buffers (hectares) Total mental health score	0.017	−0.002 to 0.036	0.079

*Adjusted for age, age squared, sex, country of birth, education, marital status, income and employment.

### Green space changes and within-person changes

**Figure 2:**
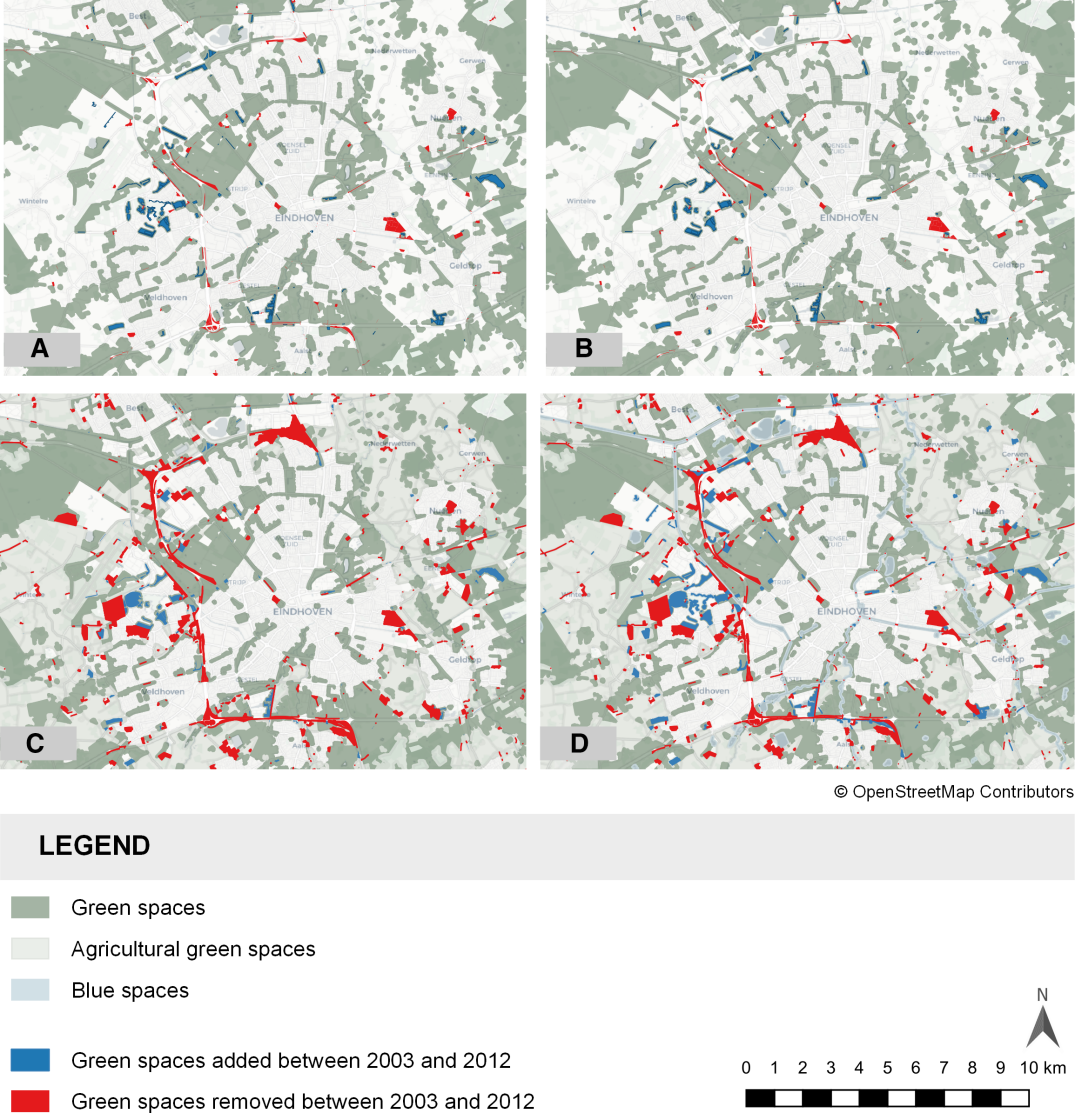
Changes in (A) green, (B) green and blue, (C) green and agricultural, and (D) green, blue and agricultural green spaces in the Eindhoven city region between 2003 and 2012.

Changes in distances to and amount of green spaces were observed over the 2003–2012 period (figure 2). Within-person changes were also observed, consisting of both increases and decreases of the total mental health score over time (table 3). It appears that more green spaces have been removed than added over this time period, resulting in more increases in the distance to the nearest green space than decreases.

**Table 3:** Within-person changes in green space and mental health between 2004 and 2014

	Decrease		No change		Increase	
	Mean	n	Mean	n	Mean	n
All participants (n=7269 person observations)						
Distance to nearest green space (m)	−131	923	0	5379	137	967
Distance to nearest green or blue space (m)	−128	847	0	5374	119	1048
Distance to nearest green or agricultural space (m)	−130	925	0	5332	133	1012
Distance to nearest green, blue or agricultural space (m)	−125	55	0	5.388	116	1076
Amount of green spaces within 300 m buffers (hectares)	−1.58	965	0	3496	1.57	942
Amount of green spaces within 500 m buffers (hectares)	−3.19	1824	0	3324	2.92	1827
Amount of green spaces within 1000 m buffers (hectares)	−8.61	2801	0	1690	7.34	2766
Total mental health score	−11.9	2955	0	1078	11.9	2808
Participants who did not relocate (n=6160 person observations)						
Distance to nearest green space (m)	−109	466	0	5182	121	512
Distance to nearest green or blue space (m)	−106	382	0	5167	103	611
Distance to nearest green or agricultural space (m)	−107	472	0	5132	121	566
Distance to nearest green, blue or agricultural space (m)	−102	390	0	5131	103	639
Amount of green spaces within 300 m buffers (hectares)	−1.06	668	0	3376	1.11	668
Amount of green spaces within 500 m buffers (hectares)	−2.04	1335	0	3232	1.97	1379
Amount of green spaces within 1000 m buffers (hectares)	−5.45	2229	0	1669	4.63	2253
Total mental health score	−11.8	2483	0	940	11.7	2349

### Fixed effects analyses

Fixed effects analyses in the total sample resulted in non-significant associations between changes in the distance to green space categories and changes in the mental health score, both for the total sample as well as for those that did not relocate during follow-up (table 4). Analyses were also performed on changes in the amount of green space within buffers and changes in mental health, but the associations were close to null (table 4). Analyses on subgroups of respondents within the city of Eindhoven and respondents within surrounding areas did not alter the results (online supplementary appendix 2).

**Table 4:** Fixed effects linear regression models regressing changes in mental health on changes in green, blue and agricultural spaces using data from 2004, 2011 and 2014 (n=8194 person observations)

Total sample n=8194 person observations	Unadjusted			Adjusted*		
	β	95% CI	P value	β	95% CI	P value
Distance to nearest green space (100 m) Total mental health score	0.18	−0.28 to 0.64	0.447	0.17	−0.28 to 0.63	0.460
Distance to nearest green or blue space (100 m) Total mental health score	0.15	−0.30 to 0.61	0.517	0.16	−0.29 to 0.61	0.486
Distance to nearest green or agricultural green space (100 m) Total mental health score	0.35	−0.16 to 0.85	0.183	0.33	−0.17 to 0.84	0.193
Distance to nearest green, blue or agricultural green space (100 m) Total mental health score	0.31	−0.18 to 0.81	0.215	0.32	−0.17 to 0.81	0.200
Amount of green spaces within 300 m buffers (hectares) Total mental health score	0.06	−0.25 to 0.36	0.715	0.06	−0.25 to 0.36	0.716
Amount of green spaces within 500 m buffers (hectares) Total mental health score	0.01	−0.10 to 0.11	0.923	0.00	−0.11 to 0.11	0.989
Amount of green spaces within 1000 m buffers (hectares) Total mental health score	0.00	−0.03 to 0.03	0.943	0.00	−0.03 to 0.03	0.894

Non-movers n=4449 person observations	Unadjusted			Adjusted*		
	β	95% CI	P value	β	95% CI	P value
Distance to nearest green space (100 m) Total mental health score	−0.40	−2.37 to 1.56	0.687	−0.36	−2.30 to 1.58	0.715
Distance to nearest green or blue space (100 m) Total mental health score	−0.28	−1.95 to 1.40	0.745	−0.25	−1.92 to 1.43	0.772
Distance to nearest green or agricultural green space (100 m) Total mental health score	−0.74	−2.89 to 1.41	0.502	−0.69	−2.83 to 1.45	0.526
Distance to nearest green, blue or agricultural green space (100 m) Total mental health score	−0.26	−2.18 to 1.65	0.789	−0.22	−2.15 to 1.70	0.819
Amount of green spaces within 300 m buffers (hectares) Total mental health score	0.25	−0.71 to 1.22	0.606	0.29	−0.69 to 1.26	0.567
Amount of green spaces within 500 m buffers (hectares) Total mental health score	0.19	−0.45 to 0.84	0.557	0.21	−0.42 to 0.83	0.518
Amount of green spaces within 1000 m buffers (hectares) Total mental health score	0.05	−0.10 to 0.19	0.526	0.05	−0.10 to 0.19	0.526

*Adjusted for time-varying confounders marital status, income and employment.

## Discussion

In this study we have linked longitudinal individual-level green space exposure data to mental health outcomes using a fixed effects approach. The present study provides evidence that the accessibility of green space is correlated with mental health, but that changes in green spaces observed during the 10-year follow-up did not lead to significant changes in mental health. The literature on this topic offers mixed results regarding the role of urban green spaces on mental health, due to variation in methodological approaches and the measurement of green spaces.^34 35^ Alcock *et al* 34 found that individuals who moved to a greener area experienced significantly better mental health while controlling for time-invariant individual-level heterogeneity and other area-level and individual-level effects within a fixed effects framework. Our study investigated the effect of a change in green space among those who did not move and found no statistically significant effects. White *et al* 6 investigated the effect of green spaces on both well-being and mental distress using a fixed effects framework and found small but significant effects for both. We were not able to replicate these results, which may be due to methodological differences. Where White *et al* 6 focused on the availability of green space defined as the percentage of green land cover within small areas, our study focused on both the accessibility and availability of green spaces within the residential environment. Analyses performed on the availability of green spaces defined as the amount of green spaces within 300/500/1000 m buffers around residential addresses did not lead to statistically significant effects in the longitudinal analyses. We did not find evidence of a change in the amount of green space within the residential environment leading to a change in mental health.

If mental health and green spaces are causally linked, then changes in green spaces in the Eindhoven area between 2004 and 2014 are not enough to produce a significant effect. Extending the follow-up of our study may mitigate this issue as we are more likely to observe changes in green spaces. However, this may also dilute the potential effect of green spaces on mental health as some of the processes believed to generate changes in mental health as a result of green space exposure may take a short time to exhibit.^36^ The current study holds value for policy makers as well, as it reflects the actual changes in the environment in the Eindhoven area between 2004 and 2014. Whereas current policies are often targeted at increasing green spaces in urban areas, our study found that overall there appeared to be as much if not more negative changes in green spaces (ie, green spaces removed) than positive changes (ie, new green spaces added). This puts into question the direction of the proposed effects. Whereas most research is focused on the question whether a greener environment will lead to better mental health, our data raise the question if a reduction in green space will also lead to worse mental health. More research is needed to explore both directions of how green space and mental health relate.

Multiple studies have also presented evidence that specific characteristics of green spaces, such as their size and quality, may influence the effect of green spaces on multiple outcomes.^28 37^ This may be especially relevant for pathways involving the accessibility of green spaces and physical activity, social interaction and health. Although our study found cross-sectional associations between green space accessibility and mental health, we did not find evidence of a causal effect of changes in the distance to the nearest green space on mental health. We were able to examine this for different types of green spaces and observed similar results. However, we were not able to control for other characteristics, such as perceived presence or quality of the green spaces. Enriching research with more relevant green space characteristics could potentially provide more insights into pathways between green space and mental health. These pathways include restoration and stress relief capacities of green spaces, which are theorised to be more related to the availability of green spaces.^4 12^ Our study found a weak association between the availability of green spaces within 1000 m buffers and mental health, but no evidence was found of an effect of changes in the availability in green space on changes in mental health.

### Strengths and limitations

The current study fills an important methodological gap by aiming to infer causal relationships between changes in green spaces and mental health that have more potential for evidence-based action. Our present study uses individual-level longitudinal green space data in a fixed effects analysis, circumventing several geographical-methodological issues associated with linking area-level exposures to individual outcomes and reducing spatial misclassification faced by area-level indicators.^19 20^ Furthermore, the fixed effects approach removes the effects of unmeasured time-invariant confounders. This is a powerful feature because these confounders are often hard to measure, and it means that fixed effects methods can alleviate omitted-variable bias.^18^ It also helps to answer a very relevant question: does a change in green space lead to a change in mental health? As most of the research on green space and mental health is cross-sectional, answering this question can help to uncover potential causal pathways between green spaces and mental health. That said, the fixed effects approach does not remove the biasing effects of time-varying confounders. To alleviate this limitation, we included relevant measured time-varying confounders in our model, but were not able to control for all potentially relevant factors, such as changes in residential density, deterioration of physical health and mobility, and changes in noise and air pollution. As fixed effects models rely solely on within-individual changes, they disregard between-individual effects and have much less power. We therefore also included a random effects model in our analyses which makes use of between-individual variance (online supplementary appendix 2). While the random effects model did not produce formal statistically significant results, it did provide estimates that were more in line with our baseline cross-sectional analysis, providing pointers that between-individual effects might be an important factor in explaining the green space–mental health relation.

As fixed effects models rely on within-individual changes, it is debatable whether the observed changes in the Eindhoven area are large enough to observe a change in mental health. As some authors advise against using change scores in longitudinal models, we also tested how baseline green space exposure influences mental health at follow-up. Including baseline green space exposure in our model did not significantly alter our results. We also tested if duration of residence in the current neighbourhood influenced our outcomes, but again this did not significantly alter the results (online supplementary appendix 2). As the green space changes in the Eindhoven area are representative of the actual changes, our analyses provide evidence that if mental health and green spaces are causally linked, then changes in green spaces in the Eindhoven area between 2004 and 2014 are not enough to produce a significant effect. More research is needed that combines the strengths of both random and fixed effects models in order to gain more insight into potential causal effects of green spaces on mental health.

The choice of land use data as the source of our exposure data was mainly based on its policy relevance, as our focus was to determine if a decrease or increase in urban green spaces could lead to better or worse mental health. Policies on urban green spaces are commonly based on land use data sets, as green space land use data represent parks and larger plots of green space that are accessible to residents. For example, the Accessible Natural Greenspace Standard, developed by Natural England, states that all residents, wherever they reside, should live within 300 m from the nearest green area.^38^ The European common indicator of local public open areas is not specifically focused on green spaces, but uses similar land use data as its basis. However, land use data do not capture fine-grained vegetation that other sources such as the Normalised Difference Vegetation Index capture.^4^ These fine-grained vegetation covers may be especially relevant for pathways considering stress reduction and attention restoration. Future research exploring pathways and underlying mechanisms between green spaces and mental health is needed. Different theorised mechanisms, such as green spaces supporting physical activity, stimulating social interactions and reducing exposure to harmful environmental stressors, may be intertwined and the direction of proposed effects is often unclear.^39^ Mediation analysis could be a valuable tool in assessing these different pathways.

One final point to consider is the specific context of our study and its external validity. The city of Eindhoven is considered to be one of the greener cities in the Netherlands compared with other large Dutch cities. As Dutch cities are considered to be very compact and dense, the spatial context of this study might not be generalisable to other cities.^40^ More research is needed that compares the effects of green spaces on mental health across different spatial contexts. Furthermore, the exposure measures in our present study were based on the residential environment, which means we were not able to control for time spent away from this residential environment. Home and neighbourhood environments are considered to be important places of ageing and a relevant spatial context for older adults.^41^ However, studies that adapt an approach where participants are tracked during the day using Global Positioning Systems (GPS) could potentially lead to more insights into how green space and mental health relate.^42 43^

## Conclusions

The introduction of more green spaces in urban settings has been widely endorsed as a method to improve both physical and mental health. While our present study finds statistically significant cross-sectional associations between accessibility to four different types of green spaces and mental health, we did not find evidence of a change in green spaces leading to a change in mental health. This has specific policy implications as gaining more insights into before-and-after effects of environmental changes has great practical relevance in public health policy. If mental health and green spaces are indeed causally linked, then changes in green spaces in the Eindhoven area between 2004 and 2014 are not enough to produce a significant effect.

**What is already known on this subject**

- Urban green spaces are often linked to better mental health and well-being through pathways such as restoration of stress and attentional fatigue, and improved physical activity.
- Exposure to green spaces has been shown to reduce chronic stress in adults living in deprived urban neighbourhoods, and self-reported mental distress has been shown to be greater in areas with lower levels of green space.

**What this study adds**

- Much of the evidence that links urban green spaces to mental health is cross-sectional, shows only short-term effects or links area-level exposures to individual-level outcomes.
- Our study links individual-level green space exposures to health outcomes with 10 years of follow-up, evaluating short-term and longer-term effects.
- By using longitudinal fixed effects methods, we circumvent methodological issues associated with cross-sectional data, therefore strengthening the evidence base of the effect of urban green spaces on mental health.
- Although we found cross-sectional evidence of an effect of green spaces on mental health, changes in green spaces did not lead to significant changes in mental health, putting into question whether more green spaces actually lead to better mental health.
