# Supplementary material for: Effect of changes in green spaces on mental health in older adults: a fixed effects analysis
Source: J Epidemiol Community Health. 2019 Oct 19;74(1):48–56. doi: 10.1136/jech-2019-212704 (PMC6929698; doi:10.1136/jech-2019-212704)
Supplement: Supplementary data [file jech-2019-212704supp001.pdf]

APPENDIX 1: MORE INFORMATION ON THE BBG DATABASE AND LAND USE CLASSIFICATION

The TOP10NL dataset is the official, national topographical representation of the Netherlands and is maintained by the Dutch mapping agency 'Kadaster'. Statistics Netherlands converts this topographical data to land use data and publishes the resulting files as open source GIS data. The 'Bestand Bodemgebruik' (BBG) is the collection of these files. The BBG dataset is generally updated every two to four years depending on funding sources and research needs. The most recent dataset is distributed by Statistics Netherlands and is reposited in the Dutch National Georegister. Historical files are distributed by the Netherlands institute for permanent access to digital research resources. These files are available through their Data Archiving and Networked Services (DANS). This platform aims to make digital research data and related outputs findable, accessible, interoperable and reusable. All historical BBG files are available through this platform free of charge.

Table 1: Green space classifications based on the land use classification of the BBG dataset

Green space categories	Corresponding BBG classifications
1. Green spaces	Parks<br>Allotment gardens<br>Dry open terrain<br>Recreational Areas
2. Green and blue spaces	Parks<br>Allotment gardens<br>Dry open terrain<br>Recreational Areas<br><br>Lakes<br>Estuaries<br>Rivers<br>Backwaters<br>Wet open terrain
3. Green and agricultural spaces	Parks<br>Allotment gardens<br>Dry open terrain<br>Recreational Areas<br><br>Agricultural areas
4. Green, blue, and agricultural spaces	Parks<br>Allotment gardens<br>Dry open terrain<br>Recreational Areas

	Lakes<br>Estuaries<br>Rivers<br>Backwaters<br>Wet open terrain<br><br>Agricultural areas

Table 2: Complete land use classification of the BBG dataset as translated by the authors

Main Group	Category	Lower Bounds (hectares)	Description
1. Traffic areas			
	10	None	Railway areas
	11	None	Road traffic areas
	12	1	Airports
2. Built environment			
	20	1	Residential areas
	21	1	Retail areas
	22	1	Public facility areas
	23	1	Social-cultural facility areas
	24	1	Business areas
3. Semi-built areas			
	30	1	Dumping grounds
	31	0.1	Junkyards
	32	0.1	Cemeteries
	33	0.5	Quarries
	34	1	Building sites
	35	1	Other
4. Recreational areas			
	40	1	Parks
	41	0.5	Sports areas
	42	0.1	Allotment gardens
	43	1	Recreational areas
	44	1	Extended stay recreational areas
5. Agricultural areas			
	50	1	Greenhouses
	51	1	General agricultural areas
6. Forests and natural terrain			
	60	1	Forests
	61	1	Open terrain (dry)
	62	1	Open terrein (mixed)
7. Backwaters			
	70	-	Lakes: IJsselmeer and Markermeer
	71	-	Closed estuaries
	72	-	Rivers: Rhine and Maas
	73	-	Border lakes
	74	1	Water reservoirs
	75	1	Recreational backwaters
	76	1	Water used for mineral extraction
	77	1	Sludge fields
	78	1	Other backwaters
8. Open waters			
	80	-	Specific open waters: Waddenzee, Eems, Dollard
	81	-	Specific open waters: Oosterschelde

	82	-	Specific open waters: Westerschelde
	83	-	North Sea
9. Borders			
	90	-	Country borders
