# Supplementary material for: Effect of changes in green spaces on mental health in older adults: a fixed effects analysis
Source: J Epidemiol Community Health. 2019 Oct 19;74(1):48–56. doi: 10.1136/jech-2019-212704 (PMC6929698; doi:10.1136/jech-2019-212704)
Supplement: Supplementary data [file jech-2019-212704supp003.pdf]

APPENDIX 3: MISSING DATA

Variable	Missing	Total	% Missing
Age			
2004	33	3,175	1.0
2011	211	3,175	11.1
2014	838	3,175	26
BMI			
2004	63	3,175	2
2011	418	3,175	13
2014	881	3,175	27.4
Education			
2004	155	3,175	4.8
2011	501	3,175	15.6
2014	864	3,175	26.8
Employment			
2004	215	3,175	6.7
2011	482	3,175	15
2014	936	3,175	29.1
Financial stress			
2004	47	3,175	1.5
2011	395	3,175	12.3
2014	915	3,175	28.4
Gender			
2004	0	3,175	0
2011	0	3,175	0
2014	0	3,175	0
Home ownership			
2004	17	3,175	0.5
2011	378	3,175	11.7
2014	896	3,175	27.8
Income			
2004	69	3,175	2.1
2011	751	3,175	23.3
2014	1,170	3,175	36.3
Marital status			
2004	54	3,175	1.7
2011	353	3,175	11
2014	848	3,175	26.3
Self-rated health			
2004	75	3,175	2.3
2011	508	3,175	15.8
2014	858	3,175	26.6

Smoking			
2004	204	3,175	6.3
2011	416	3,175	12.9
2014	863	3,175	26.8
