# Supplementary material for: Effect of changes in green spaces on mental health in older adults: a fixed effects analysis
Source: J Epidemiol Community Health. 2019 Oct 19;74(1):48–56. doi: 10.1136/jech-2019-212704 (PMC6929698; doi:10.1136/jech-2019-212704)
Supplement: Supplementary data [file jech-2019-212704supp002.pdf]

APPENDIX 2: SENSITIVITY ANALYSES

1: Linear regression models regressing total mental health on the distance to the nearest green, blue, and agricultural spaces using cross-sectional data from 2004 restricted to respondents within the suburban areas surrounding Eindhoven city (n=1,210)*

	β	95% CI	p-value
Distance to nearest green space (100m)<br>Total Mental Health Score	-0.818	-1.364 ; -0.272	0.003
Distance to nearest green or blue space (100m)<br>Total Mental Health Score	-0.647	-1.203 ; -0.091	0.023
Distance to nearest green or agricultural green space (100m)<br>Total Mental Health Score	-1.028	-1.731 ; -0.326	0.004
Distance to nearest green, blue or agricultural green space (100m)<br>Total Mental Health Score	-1.003	-1.713 ; -0.293	0.006

* adjusted for age, age squared, sex, ethnicity, education, marital status, income, and employment

2: Linear regression models regressing total mental health on the distance to the nearest green, blue, and agricultural spaces using cross-sectional data from 2004 restricted to respondents within the city of Eindhoven (n=1,910)*

	β	95% CI	p-value
Distance to nearest green space (100m)<br>Total Mental Health Score	-0.147	-0.576 ; 0.281	0.500
Distance to nearest green or blue space (100m)<br>Total Mental Health Score	-0.273	-0.714 ; 0.169	0.226
Distance to nearest green or agricultural green space (100m)<br>Total Mental Health Score	-0.076	-0.516 ; 0.363	0.734
Distance to nearest green, blue or agricultural green space (100m)<br>Total Mental Health Score	-0.203	-0.655 ; 0.250	0.380

* adjusted for age, age squared, sex, ethnicity, education, marital status, income, and employment

3: Linear regression models regressing total mental health on the distance to the nearest green, blue, and agricultural spaces using cross-sectional data from 2011 (n=2,710)*

	β	95% CI	p-value
Distance to nearest green space (100m)<br>Total Mental Health Score	-0.080	-0.468 ; 0.308	0.686
Distance to nearest green or blue space (100m)<br>Total Mental Health Score	-0.056	-0.455 ; 0.343	0.783
Distance to nearest green or agricultural green space (100m)<br>Total Mental Health Score	-0.149	-0.561 ; 0.263	0.479
Distance to nearest green, blue or agricultural green space (100m)<br>Total Mental Health Score	-0.130	-0.556 ; 0.297	0.550

* adjusted for age, age squared, sex, country of birth, education, marital status, income, and employment

4: Linear regression models regressing total mental health on the distance to the nearest green, blue, and agricultural spaces using cross-sectional data from 2014 (n=2,309)*

	β	95% CI	p-value
Distance to nearest green space (100m)<br>Total Mental Health Score	-0.122	-0.551 ; 0.306	0.576
Distance to nearest green or blue space (100m)<br>Total Mental Health Score	-0.075	-0.517 ; 0.367	0.738
Distance to nearest green or agricultural green space (100m)<br>Total Mental Health Score	-0.153	-0.605 ; 0.299	0.507
Distance to nearest green, blue or agricultural green space (100m)<br>Total Mental Health Score	-0.077	-0.547 ; 0.393	0.747

* adjusted for age, age squared, sex, country of birth, education, marital status, income, and employment

5: Random effects linear regression models regressing total mental health on the distance to the nearest green, blue, and agricultural spaces using longitudinal data from 2004, 2011, and 2014 (N=8,194 person observations)

	Unadjusted			Adjusted*		
	β	95% CI	p-value	β	95% CI	p-value
Distance to nearest green space (100m)						
Total Mental Health Score	-0.24	-0.51 ; 0.03	0.086	-0.17	-0.43 ; 0.09	0.193
Distance to nearest green or blue space (100m)						
Total Mental Health Score	-0.27	-0.55 ; 0.00	0.054	-0.19	-0.45 ; 0.08	0.169
Distance to nearest green or agricultural green space (100m)						
Total Mental Health Score	-0.25	-0.54 ; 0.05	0.097	-0.14	-0.42 ; 0.14	0.326
Distance to nearest green, blue or agricultural green space (100m)						
Total Mental Health Score	-0.28	-0.58 ; 0.01	0.062	-0.15	-0.44 ; 0.14	0.300

* adjusted for age, age squared, sex, ethnicity, education, marital status, income, and employment

6: Fixed effects linear regression models regressing changes in mental health on changes in green, blue, and agricultural spaces using data from 2004, 2011 and 2014 restricted to respondents within the suburban areas surrounding Eindhoven city (N = 2,036 person observations)

	Unadjusted			Adjusted*		
	β	95% CI	p-value	β	95% CI	p-value
Distance to nearest green space (100m)						
Total Mental Health Score	0.54	-0.43 ; 1.52	0.275	0.54	-0.42 ; 1.49	0.269
Distance to nearest green or blue space (100m)						
Total Mental Health Score	0.60	-0.33 ; 1.54	0.205	0.60	-0.31 ; 1.51	0.197
Distance to nearest green or agricultural green space (100m)						
Total Mental Health Score	0.41	-0.80 ; 1.62	0.503	0.45	-0.74 ; 1.64	0.458

Distance to nearest green, blue or agricultural green space (100m)<br>Total Mental Health Score	0.61	-0.56 ; 1.77	0.305	0.64	-0.51 ; 1.78	0.273

* adjusted for marital status, income, and employment

7: Fixed effects linear regression models regressing changes in mental health on changes in green, blue, and agricultural spaces using data from 2004, 2011 and 2014 restricted to respondents within the city of Eindhoven (N = 3,600 person observations)

	Unadjusted			Adjusted*		
	β	95% CI	p-value	β	95% CI	p-value
Distance to nearest green space (100m)<br>Total Mental Health Score	0.33	-0.47 ; 1.14	0.419	0.23	-0.57 ; 1.03	0.569
Distance to nearest green or blue space (100m)<br>Total Mental Health Score	0.07	-0.71 ; 0.86	0.854	0.00	-0.79 ; 0.78	0.997
Distance to nearest green or agricultural green space (100m)<br>Total Mental Health Score	0.24	-0.57 ; 1.06	0.559	0.16	-0.65 ; 0.96	0.703
Distance to nearest green, blue or agricultural green space (100m)<br>Total Mental Health Score	0.03	-0.77 ; 0.83	0.942	-0.03	-0.82 ; 0.76	0.939

* adjusted for marital status, income, and employment

8: Duration of residence in the current neighbourhood in 2004 (n = 3,175)

Duration of residence (years)	Frequency	%	Cumulative
1	151	4.74	4.74
2	161	5.05	9.79
3	143	4.49	14.27
4	175	5.49	19.76
5	168	5.27	25.03
6	131	4.11	29.14
7	122	3.83	32.97
8	111	3.48	36.45
9	78	2.45	38.90
10	130	4.08	42.97
11	70	2.20	45.17
12	80	2.51	47.68
13	67	2.10	49.78
14	73	2.29	52.07
15	115	3.61	55.68
>15	1400	44.32	100

9: Fixed effects linear regression models regressing changes in mental health on changes in green, blue, and agricultural spaces using data from 2004, 2011 and 2014 restricted to respondents who lived in the same neighbourhood for a maximum of 5 years (N = 2,003 person observations)

Duration of Residence <5 years<br>N = 2,003 person observations	Unadjusted			Adjusted*		
	β	95% CI	p-value	β	95% CI	p-value
Distance to nearest green space (100m)<br>Total Mental Health Score	0.00	-0.76 ; 0.75	0.996	0.53	-0.42 ; 1.49	0.269

* adjusted for marital status, income, and employment

10: Fixed effects linear regression models regressing changes in mental health on changes in green, blue, and agricultural spaces using data from 2004, 2011 and 2014 restricted to respondents who lived in the same neighbourhood for more than 5 years (N = 6,187 person observations)

Duration of Residence <5 years<br>N = 6,187 person observations	Unadjusted			Adjusted*		
	β	95% CI	p-value	β	95% CI	p-value
Distance to nearest green space (100m)						
Total Mental Health Score	0.31	-0.27 ; 0.90	0.294	0.54	-0.42 ; 1.49	0.269

* adjusted for marital status, income, and employment
